# Potential Targets and Mechanisms of Jiedu Quyu Ziyin Decoction for Treating SLE-GIOP: Based on Network Pharmacology and Molecular Docking

**DOI:** 10.1155/2023/8942415

**Published:** 2023-03-28

**Authors:** Jie Li, Run-yu Chang, Lin-feng Chen, Su-hai Qian, Rong-yun Wang, Ji-le Lan, Lin Huang, Xing-hong Ding

**Affiliations:** ^1^School of Basic Medical Sciences, Zhejiang Chinese Medical University, Hangzhou, China; ^2^College of Pharmaceutical Science, Zhejiang Chinese Medical University, Hangzhou, China

## Abstract

**Background:**

Systemic lupus erythematosus (SLE) is characterized by poor regulation of the immune response leading to chronic inflammation and multiple organ dysfunction. Glucocorticoid (GC) is currently one of the main treatments. However, a high dose or prolonged use of GC may result in glucocorticoid-induced osteoporosis (GIOP). Jiedu Quyu Ziyin decoction (JP) is effective in treating SLE and previous clinical studies have proved that JP can prevent and treat SLE steroid osteoporosis (SLE-GIOP). We aim to examine JPs main mechanism on SLE-GIOP through network pharmacology and molecular docking.

**Methods:**

TCMSP and TCMID databases were used to screen potential active compounds and targets of JP. The SLE-GIOP targets are collected from GeneCards, OMIM, PharmGkb, TTD, and DrugBank databases. R software was used to obtain the cross-targets of JP and SLE-GIOP and to perform GO and KEGG enrichment analysis. Cytoscape software was used to make the Chinese Medicines-Active Ingredient-Intersection Targets network diagram. STRING database construct protein–protein interaction network and obtain the core targets. Auto Dock Tools and Pymol software were used for docking.

**Results:**

Fifty eight targets overlapped between JP and SLE-GIOP were suggested as potential targets of JP in the treatment of SLE-GIOP. Network topology analysis identified five core targets. GO enrichment analysis was obtained 1,968 items, and the top 10 biological process, closeness centrality, and molecular function were displayed. A total of 154 signaling pathways were obtained by KEGG enrichment analysis, and the top 30 signaling pathways were displayed. JP was well bound by MAPK1, TP53, and MYC according to the molecular docking results.

**Conclusion:**

We investigated the potential targets and signaling pathways of JP against SLE-GIOP in this study. It shows that JP is most likely to achieve the purpose of treating SLE-GIOP by promoting the proliferation and differentiation of osteoblasts. A solid theoretical foundation will be provided for the future study of clinical and experimental topics.

## 1. Introduction

**Figure 1:**
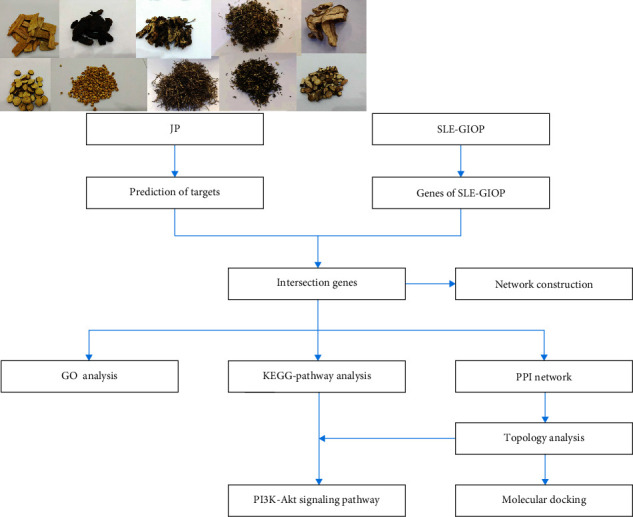
Technical route of the research based on network pharmacology.

Systemic lupus erythematosus (SLE) is a recurrent immune system disease. The cells and tissues of the patient's body are attacked by the body's own immune system, which causes facial butterfly erythema, joint pain, and damage to important organs and tissues such as kidneys. Women are more vulnerable than men, especially at childbearing age [1]. Glucocorticoid (GC) is one of the most effective immunosuppressive and anti-inflammatory drugs, which can be used to treat SLE. However, GC-related side effects are numerous and serious. Even at low doses, GC can induce osteoporosis in patients on long-term GC therapy [2]. Glucocorticoid-induced osteoporosis (GIOP) is the most common secondary osteoporosis. At the same time, epidemiological studies in premenopausal women with SLE have shown that bone mineral density in SLE patients is lower than that in women of the same age, and part of the reason for this phenomenon is the use of GC [3]. Therefore, the use of GC may not only cause GIOP in SLE patients but also increases the risk of postmenopausal osteoporosis in female SLE patients to some extent. Jiedu Quyu Ziyin decoction (JP) was created by Professor Yongsheng Fan on the basis of Shengma Biejia decoction that recorded in *Jingui Yaolue*. JP is composed of 10 traditional Chinese medicines, including Radix Rehmanniae Glutinosae (Sheng Di Huang), Carapax Amydae Sinensis (Cu Bie Jia), Rhizomn Cimicifugae (Sheng Ma), Herba Oldenlandiae Diffusae (Bai Hua She She Cao), Herba Artemisiae Apiaceae (Qing Hao), Centellae Herba (Ji Xue Cao), Radix Paeoniae Rubra (Chi Shao), Semen Coicis Lachryma-jobi (Yi Yi Ren), Fructus Citri Sarcodactylis (Fo Shou), and Radix Glycyrrhizae Uralensis (Gan Cao). It is a clinical experience prescription for the treatment of SLE based on long-term clinical experience combined with Shengma Biejia decoction. The clinical study of 147 female SLE patients showed that JP combined with western medicine could prevent and treat of GIOP in SLE (SLE-GIOP). Compared with western medicine alone, JP could significantly increase the bone mineral density of patients and had synergistic and attenuated effects [4]. By further establishing the SLE-GIOP mouse model, it is speculated that the mechanism of JP in the prevention and treatment of SLE-GIOP may be related to the protection of the hypothalamus–pituitary–adrenal axis from the inhibition of exogenous steroid hormones, the promotion of endogenous F secretion, the inhibition of PTH secretion or activity, the promotion of intestinal calcium absorption, and reduction of urinary calcium excretion [5]. However, the underlying molecular mechanisms and related pathways of treatment of SLE-GIOP are still unclear, and the relationship between therapeutic targets and pathways has not been systematically and comprehensively understood. The gradual improvement of bioinformatics and related databases has laid the foundation for a fuller understanding of network theory and systems biology. Based on this development, network pharmacology and molecular docking can help us to further explore the complex relationship between drugs and diseases. A network pharmacology approach was used in this study to obtain the effective active ingredients and core targets of JP and SLE-GIOP by extracting multiple online database information and assisting various data processing software. In order to verify the mechanism of action of JP on SLE-GIOP, we used a molecular docking method to fit the effective active ingredients and core target molecules. This provides us with a theoretical basis of JP acting on the key mechanism of SLE-GIOP. A diagram of the whole research process can be seen in Figure 1.

## 2. Materials and Methods

### 2.1. Collection of Potential Active Ingredients and Related Targets of JP

First, the components of each JP herb were extracted from Traditional Chinese Medicine Database and Analysis Platform (TCMSP) (http://lsp.nwu.edu.cn/tcmsp.php) [6] and Traditional Chinese Medicine Integrated Database (TCMID) (http://www.megabionet.org/tcmid/) [7]. Then, all compounds were initially screened using the TCMSP database based on oral bioavailability (OB ≥ 30%) and drug likeness (DL ≥ 0.18) [8]. The amount of licorice is small; therefore, setting the screening conditions for OB ≥ 60%, DL ≥ 0.36. According to the screening results, an analysis of the TCMSP database predicted the related targets of potential active components of JP. Targets' name was standardized using UniProt (https://www.uniprot.org/) [9, 10].

### 2.2. Acquisition of Known Targets for SLE-GIOP and Construction of Venn Diagrams

The SLE- and GIOP-related targets were identified in the databases GeneCards (https://www.genecards.org/), OMIM (https://omim.org/), PharmGkb (https://www.pharmgkb.org/), TTD (http://db.idrblab.net/ttd/), and DrugBank (https://www.drugbank.ca/) using the “systemic lupus erythematosus,” “SLE,” “glucocorticoid-induced osteoporsis,” and “GIOP” as the keyword. Using R 4.1.3 software and installing Venn script to obtain the target of SLE-GIOP, SLE-GIOP is a cross gene of two diseases. In order to determine the prediction target of JP related to SLE-GIOP, an R 4.1.3 software intersects the potential target of JP with the related targets involved in SLE-GIOP to obtain a Venn diagram.

### 2.3. Chinese Medicines-Active Ingredients-Intersection Targets

Cytoscape 3.8.0 software is suitable for any molecular component and interaction system and can be used to integrate and visualize molecular interaction network data [11]. The active components of each Chinese medicine and their targets and targets of SLE-GIOP were introduced into Cytoscape 3.8.0 software to construct a network diagram of “Chinese Medicines-Active Ingredients-Intersection Targets.” The large circles of the network diagram represent the active ingredients of JP, where the small circle nodes of the same color represent that they are from the same Chinese medicine, the middle square nodes represent the intersection targets, and the edges in the figure represent their interactions.

### 2.4. Construction and Analysis of Protein–Protein Interaction Network and Core Network

STRING (http://string-db.org/) is a protein–protein interaction (PPI) database, and interactions include direct and indirect associations. In addition to known interactions, it can also be used to predict interactions between proteins [12]. In order to obtain the PPI network diagram of JP and SLE-GIOP. First, the intersection targets of JP and SLE-GIOP are collated and then imported into the STRING online database. Secondly, set the interaction condition to “minimum interaction score ≥0.9” and export the results. To obtain the core target of JP against SLE-GIOP. The CytoNCA plug-in [13] in the Cytoscape 3.8.0 software was used to analyze the network topology of the PPI network results. The degree centrality (DC), betweenness centrality (BC), closeness centrality (CC), eigenvector centrality (EC), local average connectivity-based method (LAC), and network centrality (NC) of each node are calculated [14]. They are representative of the topological importance of each node based on their definitions and computational formulas [13]. The DC and BC of the node at the protein location were larger, indicating the protein had a greater significance in the network constructed [15]. Finally, other protein interaction parameters, such as CC and EC, were further used to screen core target proteins.

### 2.5. GO and KEGG Pathway Enrichment Analysis

Related R 4.1.3 software packages such as “BiocManager” were installed in advance. R 4.1.3 software was used to obtain Gene Ontology (GO) and Kyoto Encyclopedia of Genes and Genomes (KEGG) pathway enrichment analysis results and output visual images. *p*-Values were set at 0.05. GO analysis included molecular function (MF), biological process (BP), and cellular component (CC), and the top 10 results were output. KEGG analysis output the top 30 pathways with the highest significant enrichment. Finally, consulting the literature, disease-related signaling pathways were mapped using the “Pathview” software package.

### 2.6. Molecular Docking Prediction

The macromolecular proteins for molecular docking are the top three core targets in network topology analysis, and their corresponding compounds are small molecule ligands. Pub-Chem database (https://pubchem.ncbi.nlm.nih.gov/) was used to download the 2D structure of small molecule ligands. Then, use ChemBio3D Ultra 14.0.0.117 software to convert the downloaded 2D structure into a 3D structure and optimize it. After retrieving the receptor protein through the Protein Data Bank database (https://www.rcsb.org/), PyMOL 2.4.0 software was used to dehydrate and remove ligand small molecules. The protein receptor molecule was hydrogenated using Autodock Tools 1.5.7 software. Based on the position of the active site of the protein molecule and the area where it may interact with the ligand small molecule, the center coordinates and size of the box were determined [16]. AutoDock Vina 1.1.2 software was used for molecular docking, followed by analysis and visualization using PyMOL 2.4.0 software. The binding ability is evaluated by the binding energy of the receptor and the ligand. A binding energy below 0 indicates a spontaneous binding between the receptor and ligand, and the lower the value, the stronger the binding.

## 3. Results

### 3.1. Obtaining Potential Active Ingredients and Targets of JP

A total of 112 potential active ingredients were found by searching the TCMSP and TCMID databases. The potential active ingredients of SDH, CBJ, SM, BHSSC, QH, JXC, CS, YYR, FS, and GC are 3, 1, 17, 7, 22, 3, 30, 9, 5, and 15, respectively. In Table S1, we show basic information on potential active ingredients in JP. Targets of 8,015 potential active ingredients were retrieved in the TCMSP database. The number of potential targets for SDH, CBJ, SM, BHSSC, QH, JXC, CS, YYR, FS, and GC is 28, 4, 792, 556, 1,382, 615, 681, 246, 1,205, and 2,506, respectively. Finally, 1,387 effective targets were determined by removing the repetitive values of potential active ingredient targets.

### 3.2. The Acquisition of Targets at the Intersection of JP and SLE-GIOP

**Figure 2:**
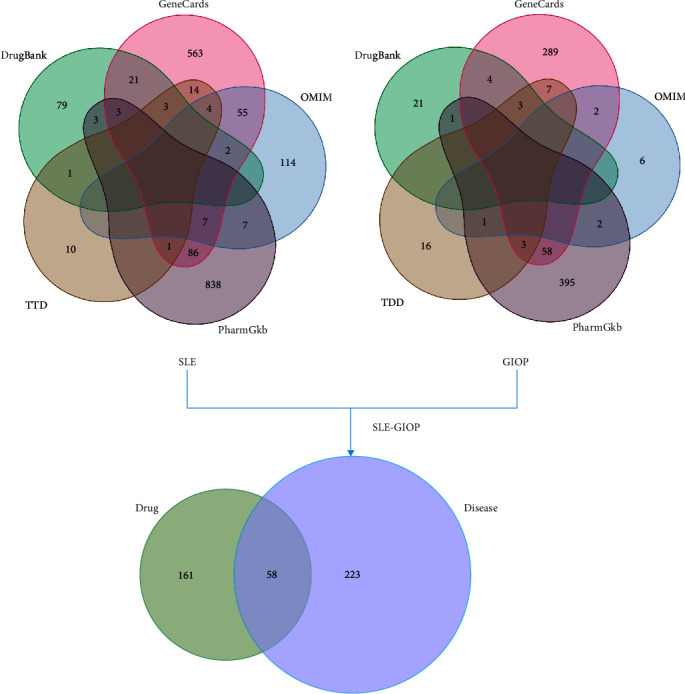
Disease (SLE-GIOP) and drug (JP) Venn diagrams.

By searching GeneCards, OMIM, PharmGkb, TTD, and DrugBank disease databases, we obtained 1,530 SLE-related targets and 527 GIOP-related targets from these databases, as shown in Figure 2. Two hundred eighty one SLE-GIOP-related cross genes were obtained after the duplication value was removed by taking the intersection. There are 58 cross-point targets between the SLE-GIOP joint targets and the potential targets of JP, as shown in Figure 2.

### 3.3. “Chinese Medicines-Active Ingredients-Intersection Targets”: Construction and Analysis

**Figure 3:**
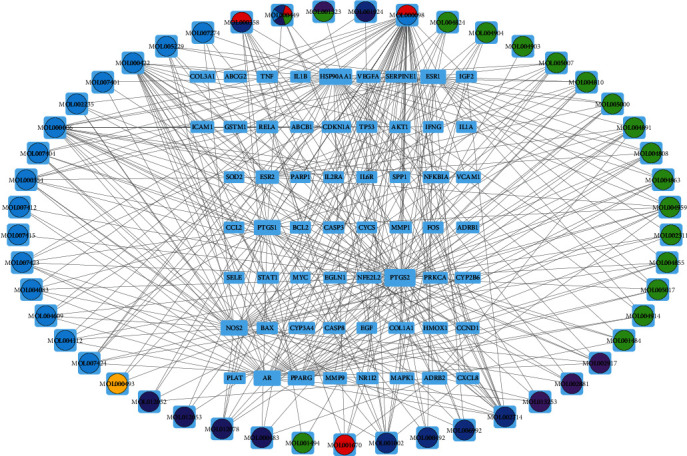
Chinese medicines-active ingredients-intersection targets network of SLE-GIOP. Starting from light green clockwise to light blue, the drugs represented by circles of different colors are: light green: gancao; light purple: foshou; dark blue: chishao; red: baihuasheshecao; dark green: yiyiren; dark purple: shengma; yellow: shengdihuang; light blue: qinghao.

Cytoscape 3.8.0 software was used to construct and analyze the Chinese Medicines-Active Ingredients-Intersection Targets network. The network diagram is shown in Figure 3. Potentially active ingredients in each herb are presented and precisely matched to SLE-GIOP disease targets. Nodes and edges reflect the specific relationship between them. The network diagram contained 58 target genes and 49 active components. The higher degree indicates that the compound plays a more critical role in the network. By further calculating the degree of the compound in the figure, the top five active compounds include MOL000098-quercetin, MOL000006-luteolin, MOL000422-kaempfero, MOL002714-baicalein, and MOL001002-ellagic acid.

### 3.4. PPI and Core Targets Network: Construction and Analysis

**Figure 4:**
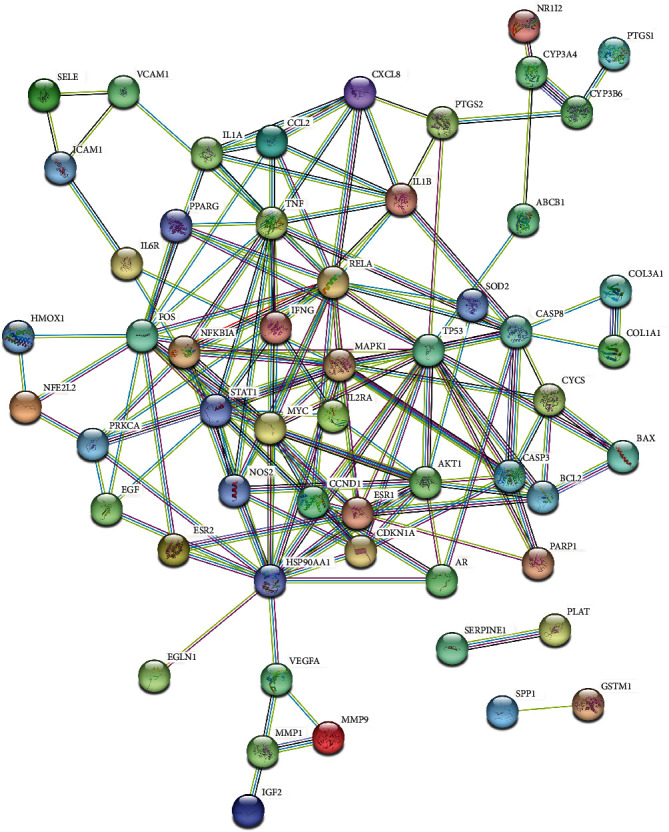
PPI network of the intersection targets of JP against SLE-GIOP.

**Figure 5:**
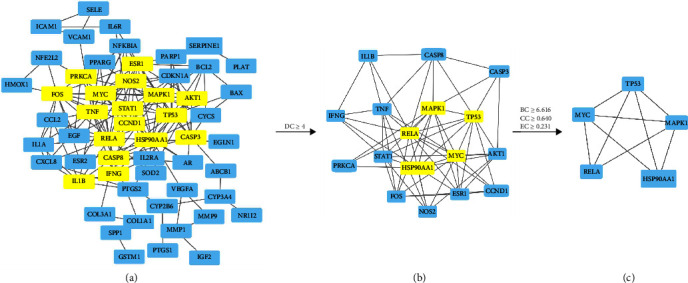
The network topology analysis of the PPI network: (a) the PPI network was generated by Cytoscape, which is comprised of 58 nodes and 156 edges; (b) the subnetwork filtered by DC is comprised of 17 nodes and 61 edges; and (c) the core network filtered by BC, CC, and EC is comprised of 5 nodes and 9 edges.

The PPI network of drug-disease intersection targets was constructed through the STRING online database platform, the result is shown in Figure 4. The core targets were screened by Cyto NCA plug-in of Cytoscape 3.8.0, and 58 nodes and 156 edges were obtained, the result is shown in Figure 5(a). For the first topological analysis, DC ≥ 4, 17 nodes and 61 edges are obtained, as shown in Figure 5(b). The second topology analysis was performed with BC ≥ 6.616 (average), CC ≥ 0.64 (average), and EC ≥ 0.231 (average) to identify the key target genes. Finally, the core target subnetwork with 5 nodes and 9 edges is obtained, and the result is shown in Figure 5(c). The top three target proteins of degree value were selected as the core target proteins in the protein network (Figure 5(c)), which were TP53 protein, MAPK1 protein, and MYC protein, respectively.

### 3.5. GO and KEGG Enrichment Analysis: Construction and Analysis

**Figure 6:**
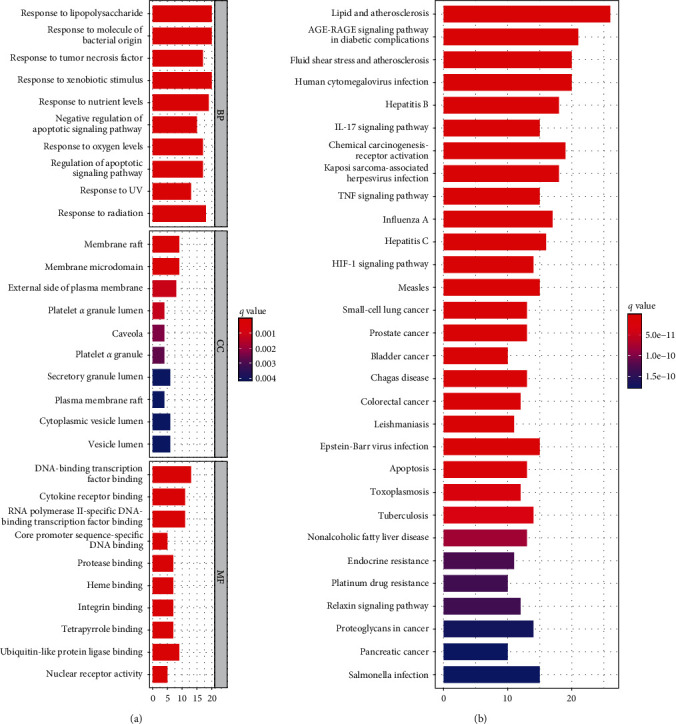
(a) Gene Ontology terms of 1,968 intersection targets. The top 10 GO functional terms were selected (*P* ≤ 0.05). BP, biological processes; CC, cellular component; MF, molecular function. (b) KEGG pathway enrichment of 154 intersection targets. The top 30 pathways were identified. Color represented *P*-value, the redder it is, the more significant enrichment.

**Figure 7:**
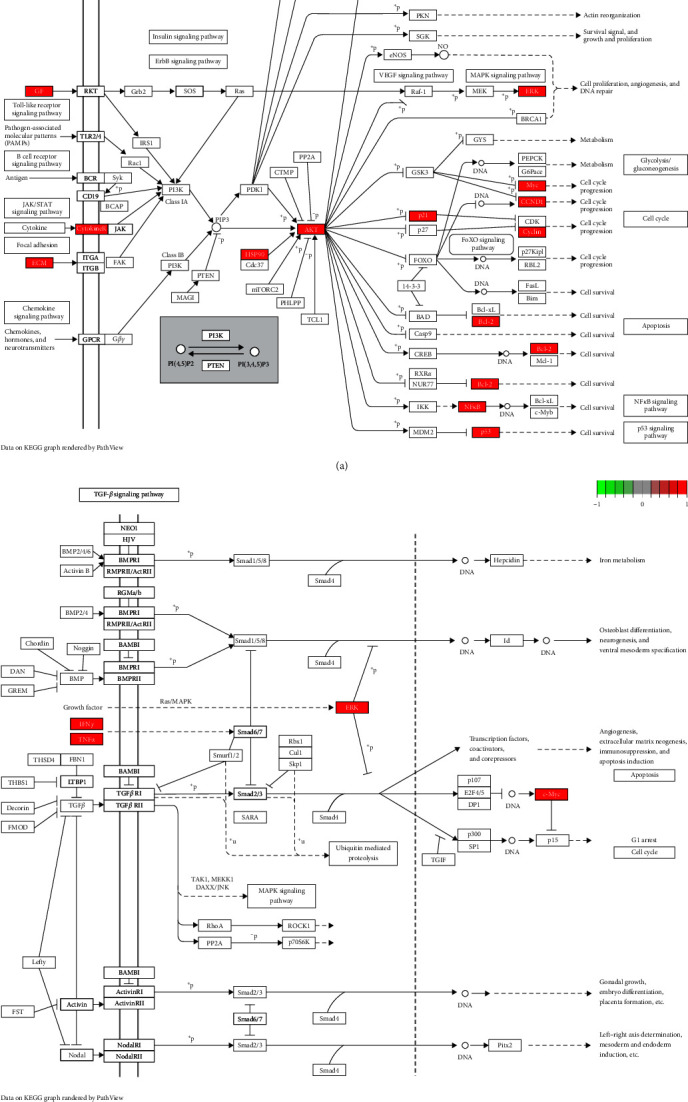
(a) PI3K-Akt signaling pathway. (b) TGF-*β* signaling pathway. Red represents the intersection targets of JP against SLE-GIOP in the signaling pathway.

R 4.1.3 software was used for GO and KEGG enrichment analysis. The results of GO enrichment analysis included BP, CC, and MF, and a total of 1,968 GO terms were identified. We selected the top 10 results of BP, CC, and MF are shown in Figure 6(a). As shown in Figure 6(a), the top three BPs are lipopolysaccharide, molecule of bacterial origin, and tumor necrosis factor. The top three CCs are membrane rafts, membrane microdomain, and external side of plasma membrane. The top three MFs are DNA-binding transcription factor binding, cytokine receptor binding, and RNA polymerase II-specific DNA-binding transcription factor binding. There are 154 signaling pathways enriched by KEGG analysis, mainly enriched in lipid and atherosclerosis, AGE-RAGE signaling pathway in diabetic complications, fluid shear stress and atherosclerosis, human cytomegalovirus infection, etc. After that, we selected the top 30 signaling pathways for visualization and are shown in Figure 6(b). After consulting the literature, two signal pathways highly related to the disease were drawn and shown in Figures 7(a) and 7(b).

### 3.6. Validation of Molecular Docking: Construction and Analysis

**Figure 8:**
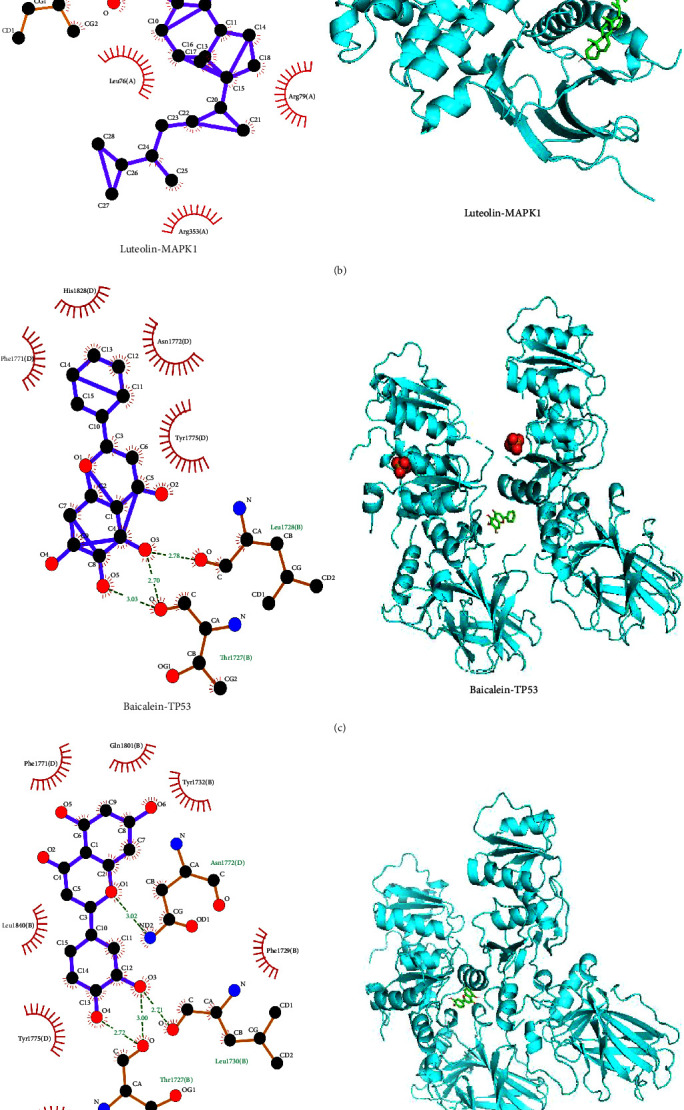
Molecular dockings of the top three core targets and their corresponding compounds: (a) MAPK1 and quercetin; (b) MAPK1 and luteolin; (c) TP53 and baicalein; (d) TP53 and luteolin; (e) MYC and quercetin.

The binding energy between molecules determines the effect of molecular docking. Lower molecular docking binding energy represents higher binding force. When the binding energy is <5 kcal/mol, the receptor and ligand have relatively good binding properties [17]. Molecular docking was used to detect the binding ability of the first three core targets in the core target network and their corresponding compounds. The docking results (2D images and 3D structures) are shown in Figure 8(a)–8(e).  Table 1 shows that the predicted potential core targets have high affinity to the effective compounds corresponding to JP, and the binding energy is almost ≤−5 kcal/mol. In addition, quercetin and MAPK1 were found to have the highest binding ability with the binding energy = −8.4 kcal/mol. Based on the above results, we can conclude that the predicted core targets and corresponding active ingredients have certain or even strong binding ability, thus verifying the credibility of the network pharmacology results.

## 4. Discussion

As a chronic recurrent disease, SLE will greatly reduce the quality of life of patients. Insufficient treatment can accelerate the damage of multiple organs and tissues. GC is the basic drug for the treatment of SLE, which is widely used to inhibit inflammation or immune system. However, high-dose and long-term use of GC will lead to a large number of patients with severe and common iatrogenic complications, namely, GIOP [18]. Based on the characteristics of multitarget and multipath effects, traditional Chinese medicine shows better curative effect on such complex diseases. JP is Professor Yongsheng Fan's empirical prescription for SLE in the long-term clinical process. The clinical efficacy of treating SLE is definite [19]. Not only does it improve the symptoms of SLE patients, but it also reduces hormone doses and is proactive in preventing and treating SLE-GIOP. However, the specific pharmacological mechanism is still unclear. The use of network pharmacology in drug research has become increasingly popular, enabling us to better understand the mechanism of action of drugs on a specific disease [20]. At the same time, our understanding of drug and target relationships can be further enhanced by verifying molecular docking. Therefore, this study used network pharmacology methods combined with molecular docking verification to explore the effective active ingredients and potential targets in JP. The potential mechanism of JP against SLE-GIOP was revealed by searching for effective active ingredients, constructing a target network, enrichment analysis of targets, and docking verification.

We used network pharmacology analysis to screen the chemical components and corresponding target genes of a single herb in the formula, and integrated the target gene information of SLE-GIOP. The results showed that JP may act on MAPK1, TP53, RELA, MYC, and HSP90AA1 through various core active components such as quercetin, luteolin, kaempferol, baicalein, and ellagic acid. Quercetin is a kind of flavonoid, which is found widely in fruits and vegetables. It can inhibit osteoclastogenesis while promoting osteogenesis, reduce oxidative stress and inflammatory response, and enhance antioxidant expression and adipocyte apoptosis. It can regulate bone metabolism through the mitogen-activated protein kinase (MAPK) signaling pathway and produce the effect of stimulating and inhibiting bone [21]. At the same time, quercetin can protect the kidney by reducing the level of proteinuria and the expression of interleukin-6 (IL-6) and tumor necrosis factor-*α* (TNF-*α*) in lupus nephritis (LN) mice [22]. Moreover, it inhibits the anti-inflammatory effect of macrophages and increases CD4 T cell activation, which can improve the symptoms of LN mice [23]. Luteolin is a plant flavonoid with antioxidant activity. Studies have shown that luteolin can promote osteoblast differentiation by regulating the ERK/Lrp-5/GSK-3*β* pathway in GIOP [24]. In a glucocorticoid-induced primary osteoporosis cell models, luteolin can not only promote the proliferation of osteoblasts but also inhibit their apoptosis [25]. Kaempferol is a dietary biological flavonoid widely found in various plants. It can exert bone protection by inhibiting adipogenesis, oxidative stress, and osteoblast apoptosis. In vitro and in vivo experimental models confirmed the osteoprotective properties of kaempferol and kaempferol-containing plants. It can exert antiosteoporosis effects by affecting multiple aspects, such as regulating bone morphogenetic protein-2 (BMP-2), MAPK, and mammalian target of rapamycin (mTOR) signaling pathways [26]. Kaempferol has also been shown to prevent and treat inflammatory diseases such as rheumatoid arthritis and SLE by increasing FOXP3 expression in Treg cells or reducing PIM1-mediated FOXP3 phosphorylation at S422 [27]. Baicalein is a lipoxygenase inhibitor that can stimulate MC3T3-E1 cells to differentiate into osteoblasts [28] and has the potential to inhibit osteoclast differentiation and induce apoptosis of mature osteoclasts [29]. In addition, the infiltration of myeloid-derived suppressor cells (MDSCs) in the kidney will lead to the acceleration and deterioration of LN. On one hand, baicalein can inhibit the expansion of MDSC, on the other hand, it can regulate the balance of Nrf2/HO-1 signal and NLRP3 expression in MDSCs to alleviate pristane-induced LN symptoms [30]. Ellagic acid, as a phenolic compound, is common in fruits, nuts, and plant extracts. Studies have shown that it can act on osteoclasts by acting on a variety of signaling pathways, such as the p38 signaling pathway downstream of RANKL, and can inhibit osteoclastogenesis and inhibit bone resorption in a dose-dependently manner, strongly protecting bone loss in vivo caused by ovariectomy-induced [31–33].

Through GO analysis of these intersectional genes, we found that JP affected SLE-GIOP through response to lipopolysaccharide, response to molecule of bacterial origin, and response to tumor necrosis factor. Combined with the literature review, KEGG enrichment analysis showed that PI3K-Akt signaling pathway and TGF-*β* signaling pathway were highly correlated with the studied diseases. Studies have found that dexamethasone can induce osteoblast apoptosis through PI3K-Akt signaling pathway [34]. In vivo and in vitro studies have shown that drugs acting on the PI3K-Akt signaling pathway can affect osteoblast differentiation, inhibit osteoblast apoptosis, and play a positive regulatory role in the osteogenic process [35, 36]. In the treatment of SLE, the accumulation of SLE lymphocytes in S and G2/M cell cycle stages is related to the increased activity of PI3K/Akt/mTOR signaling pathway [37]. HSP90 can affect the autoimmune system, which is elevated in SLE patients. 17-AAG is a HSP90 inhibitor, and the AKT/GSK3*β* signaling pathway can be downregulated by 17-AAG to inhibit the function of T lymphocytes [38]. It has been reported that the replacement of activated macrophages helps to alleviate SLE. Azithromycin can be used as an immunomodulator to alleviate SLE by promoting the alternatively activated macrophage phenotype, and the PI3K/Akt signaling pathway was involved [39]. TGF-*β* signaling pathway can affect the development of various bone types in the human body by regulating osteoblasts and osteoclasts [40]. Studies have shown that miRNA can guide mesenchymal stem cells to differentiate into osteoblasts and bone formation through TGF-*β* signaling pathway [41]. Transforming growth factor-*β*1 (TGF-*β*1) can also indirectly promote bone resorption by directly regulating the proliferation, differentiation, and survival of osteoclasts [42–44] or by regulating the expression and secretion of osteoclastogenic proteins in osteoblasts [45–47]. Meanwhile, there is evidence that SLE is associated with defective production of TGF-*β*1 by lymphocytes and its inability to regulate immunological functions [48]. Despite the fact that TGF-*β* and its ligands interact complexly, their complex interaction may represent a critical factor in regulating the tissue's response to immune injury. In most cases, the severity of proliferative glomerular lesions can be reflected by the expression of TGF-*β* [49].

The results of molecular docking showed that the predicted potential core targets bind strongly to the effective compounds corresponding to JP, with almost ≤−5 kcal/mol binding energy. In other words, it is predicted that the core targets and the corresponding active ingredients are capable of binding to them in a specific or even strong manner.

This study clarified the main potential mechanism of JP in the treatment of SLE-GIOP by screening the potential active ingredients of JP, obtaining SLE-GIOP targets, and enrichment analysis of drug and disease cross targets. The effective active ingredients of JP (such as quercetin and luteolin) can affect multiple signaling pathways related to the disease by acting on core targets (such as MAPK1 and TP53) to treat SLE-GIOP. The lower binding energy values in molecular docking indicated the possibility of interaction between the main potential active ingredients and the core targets. A few limitations, however, are present in this study. First, we only analyzed the main compounds of various traditional Chinese medicines in JP, but the interaction between compounds and the specific dosage of traditional Chinese medicine were not taken into account, which restricted the results. Second, the target and pathway information based on online database retrieval is limited by the depth and breadth of current literature research, that is, there are still unknown ways of action. Finally, theoretical research has always been to solve practical clinical problems. This research results still need further clinical experimental verification, which is also our next research focus.

## 5. Conclusion

In general, this study demonstrates preliminary evidence of JP's pharmacological effects against SLE-GIOP through network pharmacology and molecular docking. The pharmacological characteristics of JP multicomponent, multitarget, and multipathway in the treatment of SLE-GIOP were further verified by enrichment analysis. We can find a variety of effective active ingredients in JP, which can simultaneously play a role in the treatment of SLE and GIOP. The use of molecular docking preliminarily verified the interaction mode between JP active components and SLE-GIOP disease targets. This study provides a direction for further research on the mechanism of JP in the treatment of SLE-GIOP.

## Figures and Tables

**Table 1:** The optimum model for molecular docking.

Small molecule ligand	Receptor protein	Binding energy (kcal/mol)
Quercetin	MAPK1	−8.4
Luteolin	MAPK1	−8.9
Baicalein	TP53	−7.4
Luteolin	TP53	−7.3
Quercetin	MYC	−6.6

## Data Availability

The data used to support the findings of this study are included in the article.

## Supplementary Materials

Supplementary Materials All the potential pharmacologically active ingredients of JP.
